# A New Class of Quorum Quenching Molecules from *Staphylococcus* Species Affects Communication and Growth of Gram-Negative Bacteria

**DOI:** 10.1371/journal.ppat.1003654

**Published:** 2013-09-26

**Authors:** Ya-Yun Chu, Mulugeta Nega, Martina Wölfle, Laure Plener, Stephanie Grond, Kirsten Jung, Friedrich Götz

**Affiliations:** 1 Interfaculty Institute of Microbiology and Infectious Diseases Tübingen (IMIT), Microbial Genetics, University of Tübingen, Tübingen, Germany; 2 Organic Chemistry, University of Tübingen, Tübingen, Germany; 3 Munich Center for Integrated Protein Science (CiPSM) at the Department of Microbiology, Ludwig-Maximilians-Universität München, Martinsried, Germany; Harvard Medical School, United States of America

## Abstract

The knowledge that many pathogens rely on cell-to-cell communication mechanisms known as quorum sensing, opens a new disease control strategy: quorum quenching. Here we report on one of the rare examples where Gram-positive bacteria, the ‘*Staphylococcus intermedius* group’ of zoonotic pathogens, excrete two compounds in millimolar concentrations that suppress the quorum sensing signaling and inhibit the growth of a broad spectrum of Gram-negative beta- and gamma-proteobacteria. These compounds were isolated from *Staphylococcus delphini*. They represent a new class of quorum quenchers with the chemical formula *N*-[2-(*1H*-indol-3-yl)ethyl]-urea and *N*-(2-phenethyl)-urea, which we named yayurea A and B, respectively. *In vitro* studies with the N-acyl homoserine lactone (AHL) responding receptor LuxN of *V. harveyi* indicated that both compounds caused opposite effects on phosphorylation to those caused by AHL. This explains the quorum quenching activity. Staphylococcal strains producing yayurea A and B clearly benefit from an increased competitiveness in a mixed community.

## Author Summary

While studying the potential interaction of staphylococci with Gram-negative bacteria, we came across another communication system in a *Staphylococcus* species group, which consists of closely related coagulase-positive bacterial species that play a role as zoonotic pathogens. We found that these species excrete two small compounds that inhibit both the expression of QS-controlled toxins and other QS-regulated compounds as well as growth in Gram-negative bacteria. The excreted compounds, which we named yayurea A and B, were isolated from *S. delphini* and structurally characterized. They represent new bacterial products, which quench the QS regulation in a wide spectrum of Gram-negative bacteria by stimulating the LuxN-mediated phosphorylation of LuxU. Furthermore, growth of yayurea A and B producing *S. delphini* is not suppressed by respiratory toxins when co-cultured with *P. aeruginosa*. This suggests that the quorum quenchers have a function in self-protection and competitiveness in natural environments shared with Gram-negatives. Here we show one of the rare cases of inter-phylum interference between firmicutes (Gram-positive) and beta-/gammaproteobacteria (Gram-negative).

## Introduction

In many bacteria, pathogenicity is controlled and coordinated by an inter-cellular communication process named quorum sensing (QS). QS is based on the synthesis and secretion of small hormone-like molecules termed autoinducers that bind to cognate receptors. The signal-activated receptor controls directly or indirectly expression of target genes. Since the concentration of signaling molecules in liquid culture is proportional to cell density in the culture, gene expression is coordinated in response to the bacterial population density [1], [2]. In *V. harveyi*, the QS system consists of three autoinducers and three cognate receptors functioning in parallel to channel information into a shared regulatory pathway [2]. Similar to other Gram-negative bacteria, *V. harveyi* produces an AHL signal termed HAI-1, 3-hydroxy-C4-homoserine lactone [3], which binds to the membrane-bound sensor histidine kinase (LuxN). The second molecule is AI-2, a furanosyl borate diester, which binds to the periplasmic protein LuxP. The LuxP-AI-2 complex interacts with another membrane-bound sensor histidine kinase, LuxQ. The third molecule is termed CAI-1 (for cholera autoinducer-1), a long chain ketone [4], [5], which is recognized by the membrane-bound sensor histidine kinase, CqsS [6]. At low cell density, in the absence of appreciable amounts of autoinducers, the three sensors (LuxN, LuxQ, and CqsS) act as autophosphorylating kinases that subsequently transfer the phosphate to the cytoplasmic protein LuxU, which passes the phosphate to the DNA-binding response regulator protein LuxO [7], [8]. Phosphorylated LuxO represses the master regulator of QS, LuxR, via sigma factor σ^54^ and regulatory small RNAs [9], [10].

Similar to *V. harveyi*, *P. aeruginosa* coordinates the expression of nearly 10% of its genome through three hierarchically arranged QS systems, namely Las, Rhl and Pqs [11]. Each system consists of enzymes involved in autoinducer synthesis and the target regulator: LasI produces 3-oxo-C12-HSL for activation of LasR [12], RhlI produces C4-HSL for the activation of RhlR [13], [14], and the biosynthetic enzymes PqsABCDE and PhnAB produce PQS (2-heptyl-3-hydroxy-4-quinolone) for activation of PqsR [15]–[17]. QS systems are also prevalent in many other Gram-negative bacteria.

QS system is a promising target for anti-virulence therapy [1], [18]. In contrast to classic antibiotics, quorum-quenching compounds are inhibitors of bacterial virulence, rather than of bacterial growth [19]. Since the first studies on QS inhibitors, the halogenated furanones [20], more compounds have been identified [21], [22].

In contrast to Gram-negative bacteria, many Gram-positive bacteria communicate using modified oligopeptides as signals and “two-component”-type membrane-bound sensor histidine kinases as receptors. The well-studied QS system in *Staphylococcus* is the agr QS system [23]. The excreted signal is a thiolactone- or lactone-based peptide [24] (AIP, autoinducer peptide) that mediates communication with other staphylococci in a cell density dependent way [25], [26].

While studying the potential interaction of staphylococci with Gram-negative bacteria [27], [28], we came across another communication system in a *Staphylococcus* species group, named ‘intermedius group’. This group consists of closely related mainly coagulase-positive bacterial species including *S. delphini*, *S. intermedius*, *S. lutrae*, *S. pseudintermedius*, and *S. schleiferi*. They are all phylogenetically related, are zoonotic pathogens, and only rarely occur in human infections. We found that these species excrete two low molecular compounds that inhibit the expression of QS-controlled toxins and other QS-regulated compounds in Gram-negative bacteria. The excreted compounds, which we named yayurea A and B, were isolated from *S. delphini* and structurally characterized. Yayurea A and B represent new bacterial products, and were able to quench the QS regulation in a wide spectrum of Gram-negative bacteria. Furthermore, growth of yayurea A and B producing *S. delphini* is not suppressed by respiratory toxins when co-cultured with *P. aeruginosa*. This suggests that the quorum quenchers have a function in self-protection and competitiveness in natural environments shared with Gram-negatives. Here we show an example of inter-phylum interference between Firmicutes (Gram-positive) and the Gram-negative beta- and gammaproteobacteria.

## Results

### *Staphylococcus delphini* suppresses production of QS-regulated phenotypes in various Gram-negative bacteria

**Figure 1:**
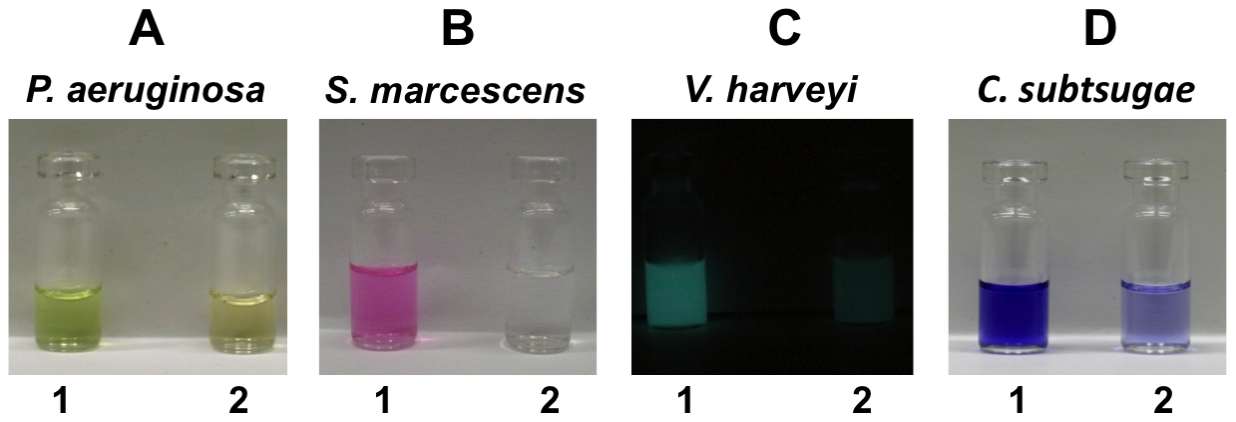
Quenching of QS-regulated pigments and bioluminescence by *S. delphini*. *P. aeruginosa* (**A**), *S. marcescens* (**B**), *V. harveyi* (**C**) and *C. subtsugae* (**D**) were each co-cultivated with *S. aureus* (1) or *S. delphini* (2) for 24 h. Pyocyanin, which is excreted by *P. aeruginosa*, was determined in the supernatant at its absorption maximum A_520 nm_. Prodigiosin, which is cell wall bound in *S. marcescens*, was ethanol-extracted from the cell pellet and determined at its absorption maximum A_534 nm_. Bioluminescence of *V. harveyi* was intensified by aeration before measuring in a bioluminescence reader. Violacein from *C. subtsugae* was quantitatively extracted with butanol and determined at its absorption maximum A_585 nm_.

Our aim was to find out if some staphylococcal species are able to suppress the QS controlled phenotypes in Gram-negative bacteria. To investigate this, we tested the ability of several staphylococcal species to inhibit pyocyanin production of *P. aeruginosa* in a co-cultivation assay, as pyocyanin production is QS controlled. We found that *S. delphini* DSMZ 20771 completely inhibited pyocyanin production over 24 h co-cultivation with *P. aeruginosa* PAO1, while *Staphylococcus aureus* showed no such activity (Fig. 1A). This led us to investigate if *S. delphini* could also suppress QS-controlled phenotypes in other Gram-negative bacteria such as the QS-regulated prodigiosin production in *Serratia marcescens* [29]; bioluminescence in *Vibrio harveyi* [7], [30]; or violacein production in *Chromobacterium subtsugae*, a pathogen of potato beetles [31]. Indeed, in co-cultivation studies with these Gram-negative bacteria, *S. delphini* did also suppress prodigiosin and violacein production as well as bioluminescence, while *S. aureus* did not (Fig. 1BCD). Sterile filtered culture supernatant of a 24 h *S. delphini* culture had the same QS-inhibiting effect as the co-culture, indicating that the QS-inhibiting compound(s) were excreted. The supernatants of *S. aureus* and *S. epidermidis* caused no effect.

### Structural analysis of the QS-inhibiting compounds from *S. delphini*

The QS inhibitors were isolated from the supernatant of an overnight culture of *S. delphini* DSMZ 20771. Further purification revealed that the supernatant contained two compounds with different retention times (R_t_) in HPLC and distinct UV spectra. We named the two compounds yayurea A and B.

**Figure 2:**
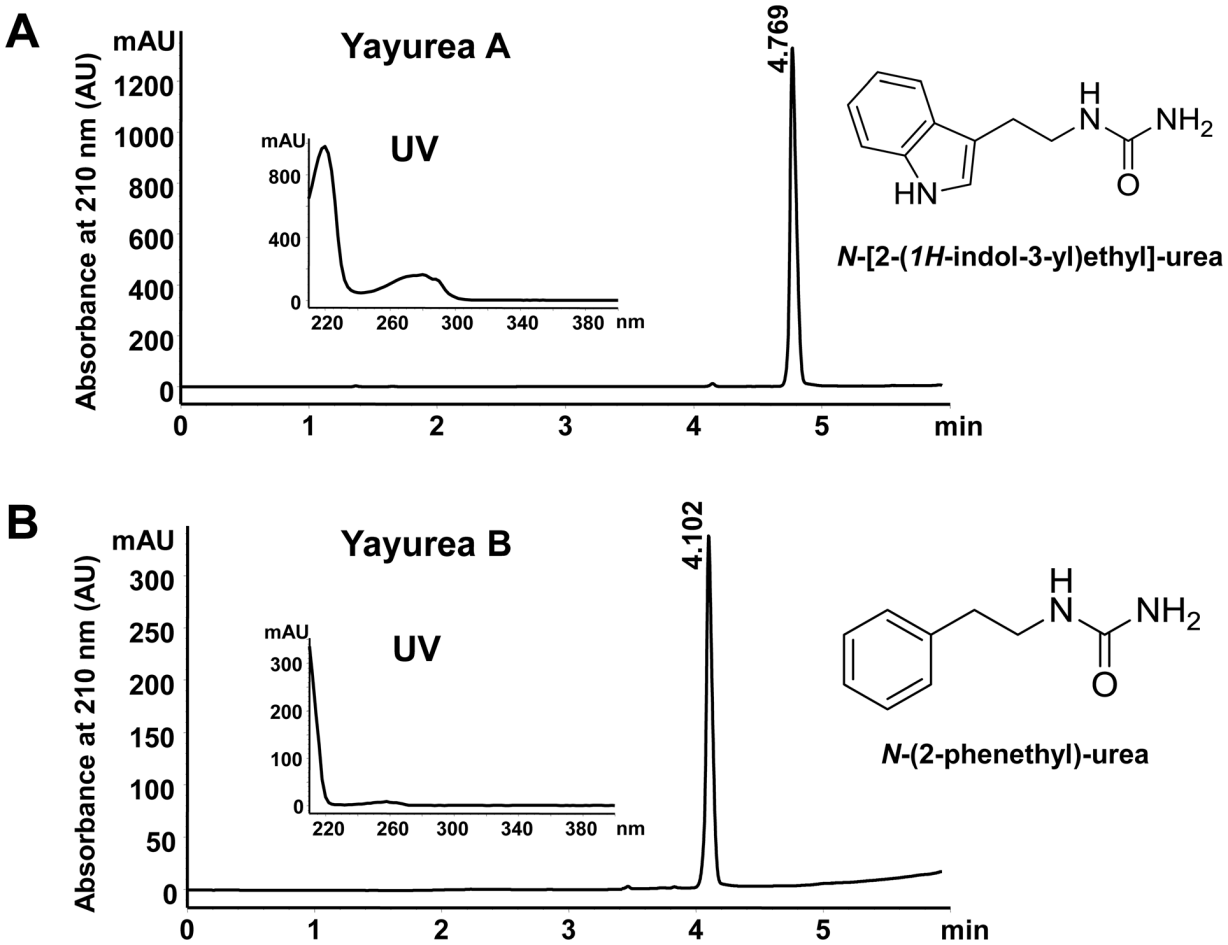
RP-HPLC profile, UV-spectrum and structures of the two QS-inhibitors purified from *S. delphini*. (**A**) QS-inhibitor, *N*-[2-(1H-indol-3-yl)ethyl]-urea (yayurea A). (**B**) QS-inhibitor, *N*-(2-phenethyl)-urea (yayurea B). RP-HPLC was carried out on an Agilent 1200 and Waters xBridge C18, 5 mm, 4.6×150 mm column; compounds were eluted with a 15 min linear gradient of 0.1% phosphoric acid to acetonitrile at a flow rate of 1.5 ml/min.

Yayurea A (indole-ethylurea) was isolated as a brownish solid, revealed an ion peak at m/z = 161 ([M+H]^+^) in ESI-MS, and showed the molecular formula, C_10_H_13_N_2_, in FT-ICR-MS analysis. The 1*H*-indole-3-ethylamine moiety was deduced from GC-EI-MS (R_t_ = 29.0 min). ^1^H-NMR spectra showed a characteristic singlet at δ_H_ = 8.55 ppm. The remaining signals were assigned to 2-(3-indoyl) ethylamine. The ^13^C-NMR spectrum and additional HSQC experiments displayed signals pointing to a carbonyl group at δ = 170.4 ppm, five methin groups, and two methylene groups to reveal a urea moiety. Additionally, 2D NMR experiments supported the structure of yayurea A as *N*-[2-(1H-indole-3-yl) ethyl]-urea (Fig. 2A).

Yayurea B (phenethylurea) was obtained as a colorless solid. A preliminary molecular formula, C_8_H_10_N, was deduced from the FT-ICR-MS spectrum which showed an ion at m/z = 121 ([M+H]^+^). GC-EI-MS provided a signal at R_t_ = 15.0 min pointing to 2-phenethylamine. The ^1^H-NMR again showed a set of aromatic protons and a singlet at δ_H_ = 8.54 ppm, while the ^13^C-NMR displayed a signal at δ_H_ = 170.4 ppm (C = O) and five aromatic and 4 aliphatic protons. In summary, the structure of *N*-(2-phenethyl)-urea was assigned for yayurea B (Fig. 2B). UV-Absorption maxima emphasized the phenyl- (λ = 260 nm) and indole chromophores (λ = 225 nm and λ = 280 nm respectively) of the yayureas. Comprehensive physicochemical characteristics and mass spectra of yayurea A and B are shown (Table 1 and Figure S1). In the meantime, yayurea A and B could also be chemically synthesized and their identity confirmed by mass spectral and NMR analyses (Wölfle *et al.* manuscript in preparation). Both the synthesized and the natural compounds revealed the same chemical properties and the same QS-quenching activity in Gram-negative bacteria (data not shown).

**Table 1:** Physical data for yayurea A and B.

	*N*-[2-(*1H*-indol-3-yl)ethyl]-urea (yayurea A)	*N*-(2-phenethyl)-urea (yayurea B)
**Formula**	**C_11_H_13_O_1_N_3_** (203.24 g/mol)	**C_9_H_11_O_1_N_2_** (163.20 g/mol)
**Melting point**	240.8°C	214.9°C
**R_f_-values**	0.10 (CHCl_3_/MeOH 9∶1) 0.63 (MeOH/H_2_O 7∶3)	0.28 (CHCl_3_/MeOH 9∶1) 0.94 (MeOH/H_2_O 7∶3)
**^1^H NMR**	(600 MHz, MeOH-*d_4_*) d 3.11 (t, J = 7.0, 7.4 Hz, 2H), 3.22 (t, J = 7.1, 7.3 Hz, 2H), 7.04 (dd, J = 7.4, 7.5 Hz, 1H), 7.13 (dd, J = 7.7, 7.9 Hz, 1H), 7.17 (s, 1H), 7.37 (d, J = 8.2 Hz, 1H), 7.57 (d, J = 7.9 Hz, 1H), 8.55 (s, 1H).	(600 MHz, MeOH-*d_4_*) d 2.95 (t, J = 7.4, 8.1 Hz, 2H), 3.16 (t, J = 7.4, 8.1 Hz, 2H), 7.28 (m, 3H), 7.35 (m, 2H), 8.54 (s, 1H).
**^13^C NMR**	(150 MHz, MeOH-*d_4_*) d 24.9, 41.5, 110.6, 112.7, 119.0, 120.2, 122.9, 124.4, 128.4, 138.5, 170.5.	(150 MHz, MeOH-*d_4_*) d 34.9, 42.1, 128.4, 129.9, 130.2, 138.1, 170.2.
**MS (ESI)**	(Positive ions) *m/z* (%) [M+2H-CONH_2_]^+^ 161.11.	(Positive ions) *m/z* (%) [M+2H-CONH_2_]^+^ 122.10.

### Suppression of QS-regulated respiratory toxins protects *S. delphini* from killing by *Pseudomonas aeruginosa*

**Figure 3:**
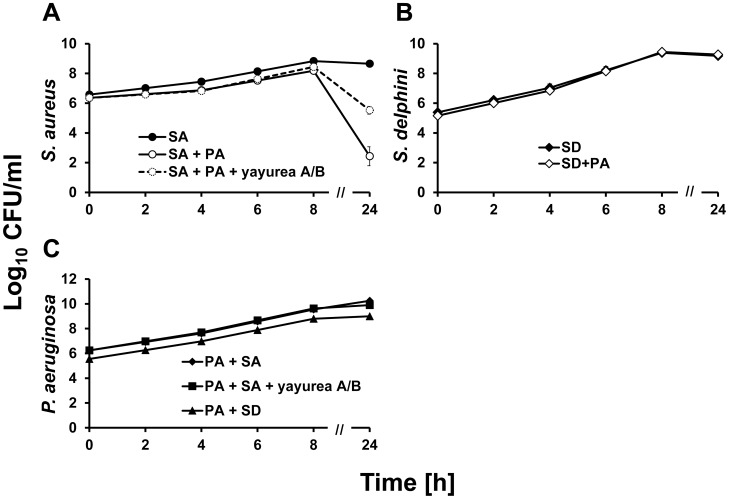
Survival of staphylococcal strains in mixed culture with *P. aeruginosa*. (**A**) CFU of *S. aureus* alone (SA) and in co-culture with *P. aeruginosa* (SA+PA). For the protection test, yayurea A (100 µg/ml) and B (900 µg/ml) were added to the mixture of *S. aureus* and *P. aeruginosa* (SA+PA+yayurea A/B). (**B**) CFU of *S. delphini* alone (SD) and in co-culture with *P. aeruginosa* (SD+PA). (**C**) CFU of *P. aeruginosa* SH1 co-cultured with *S. aureus* (PA+SA), yayureas (PA+SA+yayurea A/B) or *S. delphini* (PA+SD). Values represent the means of three independent experiments. Bars indicate mean standard deviation, SD.

*P. aeruginosa* produces various QS-controlled respiratory toxins such as pyocyanin and hydrogen cyanide [32], [33], which kill *S. aureus* [27]. If yayurea A and B repress the production of QS-regulated pyocyanin or hydrogen cyanide, one would expect that *S. delphini* survives better in a co-culture with *P. aeruginosa* than, for example, *S. aureus*. Indeed, co-cultivation studies with *S. aureus* (non-producer) or *S. delphini* with *P. aeruginosa* SH1 revealed that the viability (CFU) of *S. aureus* significantly decreased in the stationary growth phase, most likely due to the respiratory toxins produced by *P. aeruginosa* (Fig. 3A), while that of *S. delphini* was uninfluenced (Fig. 3B). The addition of yayurea A and B to the mixed *S. aureus* and *P. aeruginosa* culture protected *S. aureus* from killing in the stationary phase (Fig. 3A). Furthermore, the CFU of *P. aeruginosa* SH1 was unaffected while co-cultured with *S. delphini* or *S. aureus*, indicating that none of the two staphylococcal species was able to kill *P. aeruginosa*. Co-cultivation of *P. aeruginosa* with *S. aureus* in the presence of yayureas (100 µg/ml yayurea A and 900 µg/ml yayurea B) had also no effect on viability of *P. aeruginosa* (Fig. 3C). Other tested *Pseudomonas* strains PAO1 and DSMZ 50071 showed similar results (data not shown). All in all, these results showed that production of yayurea A and B enables staphylococci to coexist with Gram-negative bacteria in a mixed community.

### Yayurea A and B are mainly produced in stationary growth phase of *S. delphini*

**Figure 4:**
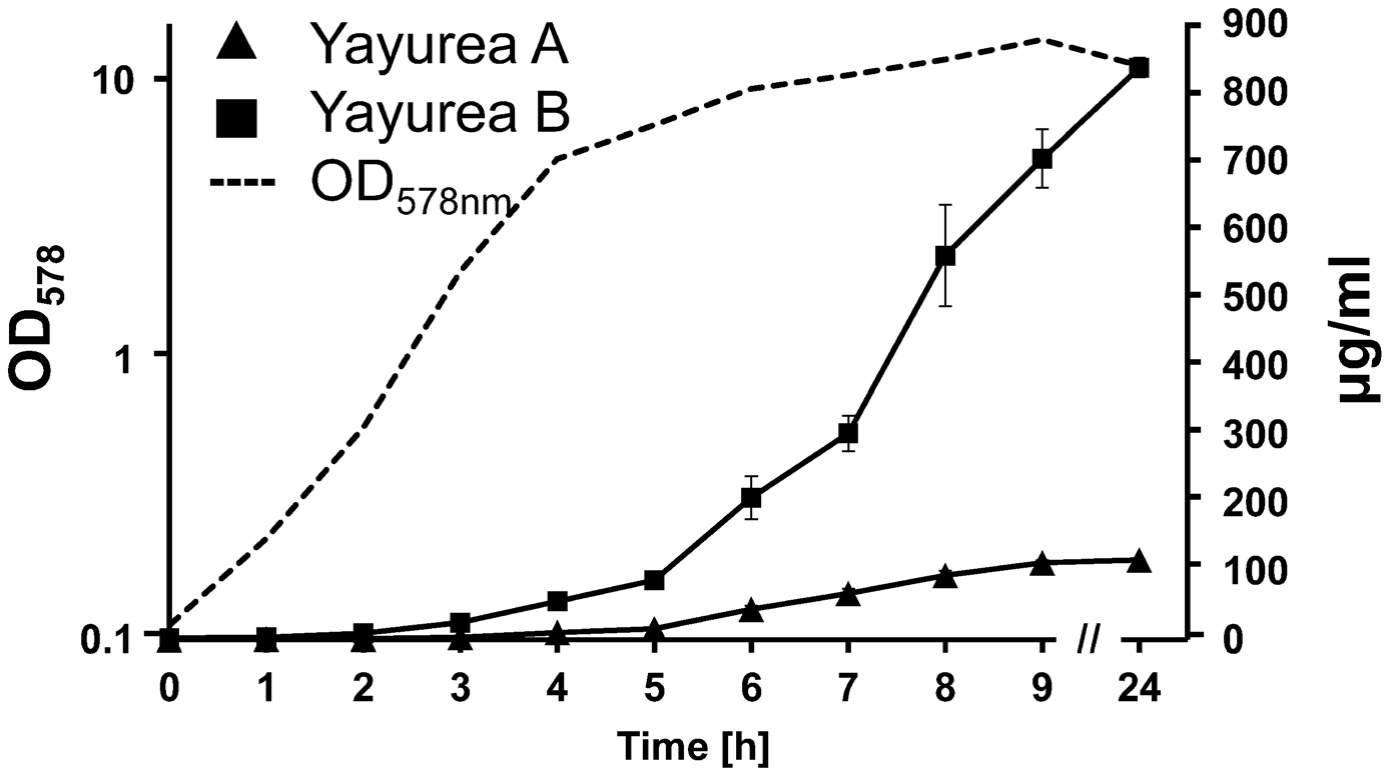
Quantification of yayurea A and B production in relation to growth. *S. delphini* was grown in TSB at 37°C. Supernatant was collected and OD_578_ was measured hourly for the first 9 h and after 24 h. Amounts of yayurea A and B in supernatants were quantified by triplicate HPLC measurements. Bars indicate mean standard deviation, SD.

We followed the production of yayurea A and B over 24 h in the supernatant *S. delphini* grown in TSB. The amount of produced yayurea A and B was determined by HPLC-analysis; peak integration was correlated with standard yayurea A and B. The production of both compounds started at the transition of exponential to stationary growth phase (after approximately 4 h) and increased rapidly for the next 5 h; after 24 h little more was produced (Fig. 4). The production kinetics is reminiscent of a QS controlled expression. Both compounds were produced in amazingly high concentrations: yayurea A reached concentration of 120 µg/ml and yayurea B even 900 µg/ml. This high concentration is entirely sufficient to suppress QS-systems in Gram-negative bacteria as can be seen below.

### Yayurea A is more active than yayurea B in inhibiting various QS-controlled traits in Gram-negative bacteria

**Figure 5:**
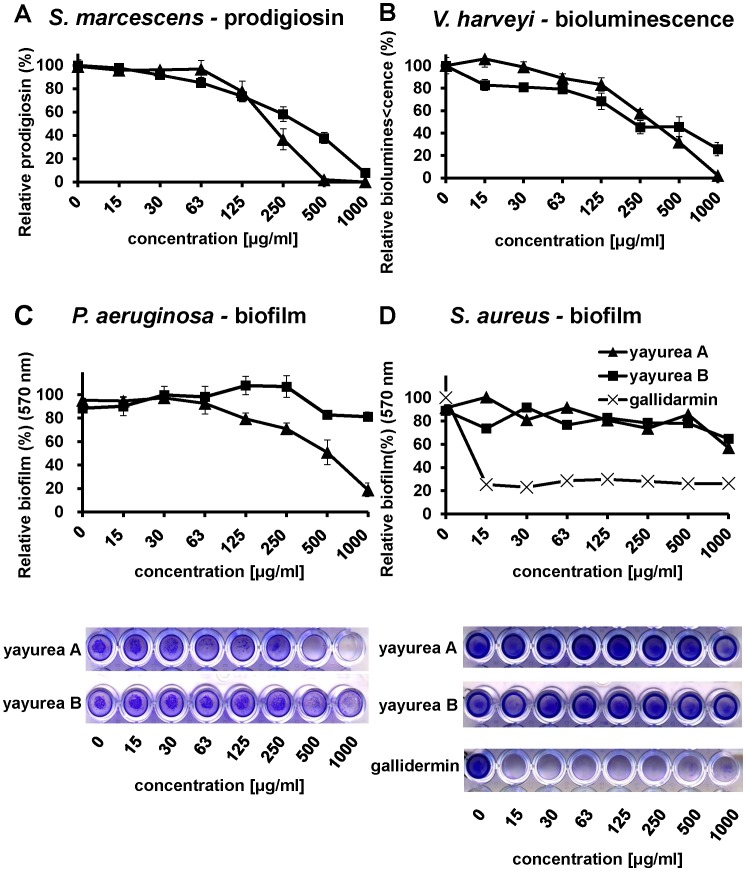
Concentration-dependent inhibition of QS-regulated phenotypes in Gram-negative bacteria. (**A**) Prodigiosin production in *S. marcescens*. Cells were grown in MB medium with serial dilutions of yayurea A or B at 28°C. Relative prodigiosin production was calculated as the ratio between prodigiosin content (absorbance at 534 nm) and cell density (absorbance at 600 nm). (**B**) Bioluminescence in *V. harveyi*. Cells were grown in marine broth with serial dilutions of the compounds at 28°C for 24 h. Relative luminescence units were normalized by the cell density. (**C**) Biofilm formation of *P. aeruginosa*. Cells were grown in LB with serial dilutions of yayurea A or B at 37°C for 24 h. (**D**) Biofilm formation of *S. aureus*. Cells were grown in TSB with serial dilutions of yayurea A, B, or gallidermin (positive control) at 37°C for 24 h. Biofilm cell layer was visualized by crystal violet staining and measured at 590 nm. Microtiter plates presented are representative of at least three independent sets of experiments. Bars indicate standard deviation of the mean, SD.

**Figure 6:**
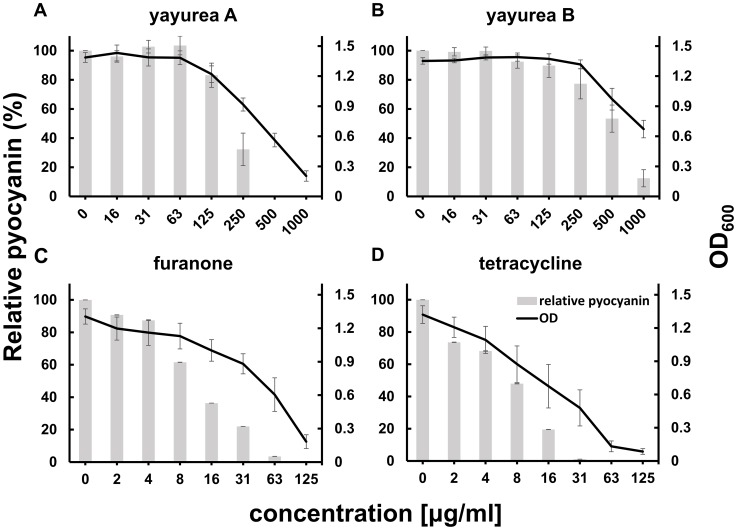
Concentration-dependent inhibition of pyocyanin production and growth of *P. aeruginosa*. *P. aeruginosa* PAO1 was grown in LB at 30°C with serial dilutions of yayurea A (**A**), yayurea B (**B**), furanone (**C**) and tetracycline (**D**). Relative pyocyanin production was calculated as the ratio between pyocyanin content and cell density (absorbance at 600 nm). Values represent the means of three independent experiments. Bars indicate standard deviation of the mean, SD.

Purified yayurea A and B inhibited QS-regulated factors in Gram-negative bacteria in a dose-dependent manner. We tested prodigiosin production in *S. marcescens*, bioluminescence in *V. harveyi*, and pyocyanin production in *P. aeruginosa* (Fig. 5 and 6).

Yayurea A and B inhibited production of prodigiosin in *S. marcescens* in a dose-dependent way. Inhibition already started at low concentrations (15 µg/ml) and increased with increasing concentrations of yayurea A or B. At 250 µg/ml, prodigiosin production was inhibited at 60% (yayurea A) and 40% (yayurea B). At a concentration of 1000 µg/ml, prodigiosin production was completely inhibited by yayurea A, and to approximately 70% by yayurea B (Fig. 5A). At 500 µg/ml, bioluminescence *of V. harveyi* was inhibited by yayurea A and B by 99% and 76% respectively (Fig. 5B). We also investigated whether yayurea A and B inhibited biofilm formation in *P. aeruginosa* and *S. aureus*. Again yayurea A quite efficiently inhibited biofilm formation in *P. aeruginosa*, while yayurea B was less effective (Fig. 5C). In contrast to gallidermin, a good biofilm inhibitor in staphylococci [34], both yayureas showed no biofilm-inhibiting effect with *S. aureus* (Fig. 5D).

We noticed that high dose of yayurea A and B (especially yayurea A) inhibit the growth of Gram-negative bacteria. We used *P. aeruginosa* as an example to verify the effect of growth and QS inhibition by yayurea A and B, and used the antibiotic tetracycline and the well-known QS-inhibitor furanone [35] as controls (Fig. 6). For QS inhibition, around 50% of the yayurea A and B concentration was necessary compared to that needed for growth inhibition (Fig. 6 A, B). Furanone revealed a similar correlation of growth and QS inhibition; the concentration (31 µg/ml) that inhibited 40% of growth inhibited 80% of QS (Fig. 6C). In contrast to yayurea and furanone, tetracycline inhibited growth and QS almost linearly (Fig. 6D). Besides *P. aeruginosa*, yayureas affect also growth of *S. marcescens*, *V. harveyi*, and *V. cholerae* (Figure S2); however, growth of *E. coli* was not affected. All staphylococcal species tested, such as *S. aureus*, *S. carnosus*, *S. delphini* or *S. schleiferi*, are resistant to yayurea A and B, independently whether they are producing these compounds or not.

### Yayurea A and B are perceived by the AHL-receptor LuxN of *Vibrio harveyi*

**Figure 7:**
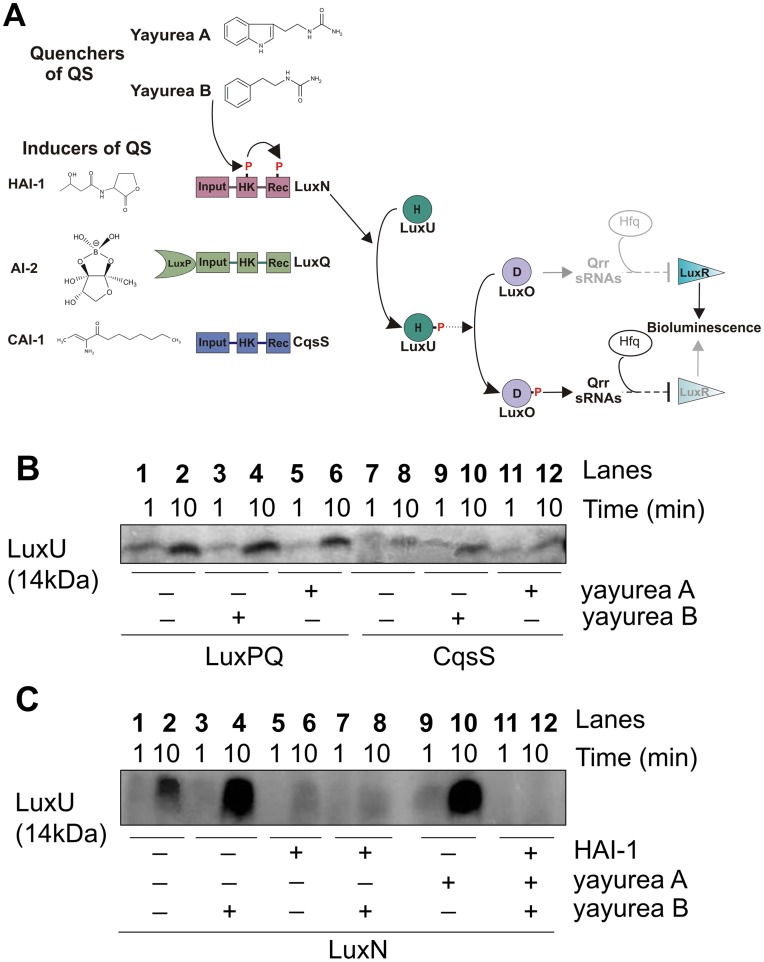
Yayurea A and B are perceived by *V. harveyi* LuxN receptor. (**A**) Schematic representation of the QS phosphorelay in *V. harveyi*. In the absence of autoinducers (HAI-1, AI-2 and CAI-1) at low cell density, each of the three receptors, LuxN, LuxQ and CqsS, respectively, autophosphorylates at a conserved histidine of their histidine kinase domain (HK). The phosphoryl group is first transferred to the receiver domain (Rec) of the receptor kinase and then to the HPt protein LuxU. LuxP is a periplasmic binding protein. P denotes phosphorylation sites. Upon perception of the autoinducers at high cell density, autophosphorylation of the receptors and the subsequent phosphosphrylation cascade is inhibited. Yayurea A and B stimulated the phosphorylation of the cascade via LuxN. (**B**) LuxPQ and CqsS mediated phosphorylation of LuxU in the presence of yayurea A and B. (**C**) LuxN mediated phosphorylation of LuxU in the presence of yayurea A and B and HAI-1. LuxQ, CqsS, LuxN-bearing membrane vesicles and LuxU, were incubated with 100 µM [γ-^32^P] ATP. The effect of yayurea A, B and HAI-1 (**C**) on the initial rate of LuxU phosphorylation was evaluated. Each reaction was sampled and stopped at two different time points: after 1 and 10 minutes. Final concentrations were 20 µM for HAI-1, 1.1 mM for yayurea A and 1.3 mM yayurea B, which reflects the *in vivo* situation. Absence of HAI-1 or yayurea A or B is indicated by “−” and presence by “+”.

To gain insight into the molecular mechanism behind why yayurea A and B cause a decrease in bioluminescence of *V. harveyi*, we performed *in vitro* phosphorylation assays of the corresponding signaling proteins. The full-length hybrid kinases LuxN, LuxQ, CqsS (tagged with 6 histidine residues) were heterologously expressed in an *E. coli* strain that lacks the F_1_/F_o_-ATPase (to prevent ATP degradation during phosphorylation experiments), and inverted membrane vesicles prepared from this strain were directly used for the phosphorylation experiments. Then we tested the effect of yayurea A and B on the LuxN, LuxQ [in interplay with LuxP (LuxPQ)], and CqsS-mediated time-dependent phosphorylation of the HPt protein LuxU (Fig. 7A). Yayurea A and B had no effect on LuxPQ or CqsS-mediated phosphorylation of LuxU (Fig. 7B). However, they significantly stimulated the LuxN-mediated phosphorylation of LuxU in comparison to the control (Fig. 7C, compare lane 2 with lanes 4 and 10). LuxN is the sensor for the AHL autoinducer *N*-(3-hydroxybutyryl)-homoserine lactone (HAI-1) (Fig. 7A). It is known [36] that the presence of HAI-1 inhibits the autophosphorylation activity of LuxN, thus decreasing the level of phospho-LuxU (Fig. 7C, compare lanes 2 and 6).

Yayurea A and B caused the opposite effect, leading to an increase in the level of phospho-LuxU, which explains the decrease in bioluminescence when *V. harveyi* is exposed to these compounds. Under our test conditions, autoinducer HAI-1 was dominant in relation to yayurea A or B (Fig. 7C, compare lane 4 with lanes 8 and 12). Taken together these data show that the receptor kinase LuxN of *V. harveyi* specifically recognizes yayurea A and B. It is still unclear whether yayurea A and B also bind to the HAI-1 binding site or whether LuxN contains an independent binding site for these new compounds.

### Phylogenetic position of yayurea A and B producing species in the *Staphylococcus* taxa

We tested a number of staphylococcal species representatives (listed in Table 2) by co-cultivation with *P. aeruginosa* for suppression of pyocyanin production. Only five species exerted such an activity: *S. delphini*, *S. intermedius*, *S. lutrae*, *S. pseudintermedius*, and *S. schleiferi*, and they all produced yayurea A and B as determined by HPLC analysis. Based on 16S rRNA- and *multilocus sequence typing* (MLST) the species are phylogenetically related and summarized in the ‘intermedius group’ [37] (Figure S3). Typically, the species are coagulase positive (with exception of *S. schleiferi* subsp. *schleiferi*), and oxidase negative. Interestingly, they all colonize various animals, and many represent zoonotic pathogens [38], [39]. None of the other listed staphylococcal species (even not the next related *S. hyicus*, *S. chromogenes* or *S. muscae*) inhibited QS-system in Gram-negative bacteria or produced yayurea A and B.

**Table 2:** Strains tested for Q-quenching activity and yayurea A and B production.

Strains	QS-inhibition
*S. aureus RN4220*	−
*S. aureus SA113*	−
*S. aureus 8325-4*	−
*S. aureus RN1*	−
*S. aureus HG001*	−
*S. aureus HG002*	−
*S. aureus HG003*	−
*S. aureus Newman*	−
*S. arlettae* DSMZ 20672T	−
*S. carnosus*TM300 DSMZ 20501	−
*S. capitis* subsp. *capitis* LK499 ATCC 27840	−
*S. caprae* DSMZ 20608T	−
*S. chromogenes* DSMZ 20454T	−
*S. cohnii* subsp. *cohnii* DSMZ20260	−
*S. condiment* DSMZ 11674T	−
*S. delphini* DSMZ 20771	+++
*S. epidermis* ATCC14990	−
*S. equorum* subsp. *equorum* DSMZ 20674T	−
*S. gallinarum* DSMZ 20610T	−
*S. muscae* DSMZ 7068T	−
*S. haemolyticus* CCM2737	−
*S. hominis* DSMZ 20328	−
*S. hyicus* NCTC 10350	−
*S. intermedius* CCM 5739	+
*S. lentus* DSMZ 20352T	−
*S. lugdunesis* ATCC 43809	−
*S. lutrae* DSMZ10244T	+
*S. pasteuri* ATCC 51129	−
*S. pseudintermedius* ED99	+++
*S. saprophyticus* subsp. *saprophyticus* DSMZ 200229	−
*S. schleiferi* subsp. *coagulans* ATCC49545	++
*S. schleiferi* subsp. *schleiferi* DSMZ 4807	+++
*S. simulans* MK148 ATCC 27848	−
*S. warneri* DSMZ 20316T	−
*S. xylosus* DSMZ 20266	−

(+), Species that produce yayurea A and B.

## Discussion

In this study, we found that some staphylococcal species excrete two novel compounds, yayurea A and B that interfere with the QS system of diverse Gram-negative bacteria. We tested 24 staphylococcal species with respect to production of yayurea A and B by co-cultivation with Gram-negative bacteria. The results showed that only five species (*S. delphini* [40], *S. intermedius* [41], *S. pseudintermedius* [42], *S. lutrae* [43], and *S. schleiferi* [44]) produced these compounds and were able to inhibit QS-regulated markers of Gram-negative test bacteria. All five species are naturally associated with various animals, where they act as zoonotic pathogens. They are only rarely associated with human infections. It is remarkable that these species are clustered in various phylogenetic trees, based on either 16S rRNA [45], [46], on thermonuclease (*nuc*) sequences [47], on major autolysin (atl) sequences [48], or on multilocus sequence typing (MLST) [37]. The five species form a phylogenetic cluster, termed “intermedius group” (Figure S3).

To gain insight into the mode of QS quenching by yayurea A and B, we performed *in vitro* phosphorylation assays with the autoinducer receptors LuxN, LuxQ, CqsS (Fig. 7A). Yayurea A and B had no effect on LuxPQ or CqsS-mediated phosphorylation of LuxU. However, they significantly stimulated the LuxN-mediated phosphorylation of LuxU (Fig. 7B–C), suggesting that they interact with LuxN and cause LuxU activation. While the LuxN autoinducer HAI-1, an AHL, inhibits the autophosphorylation of LuxN, consequently leading to a decrease in LuxU-phosphorylation, yayurea A and B caused the opposite effect by increasing LuxU phosphorylation. Thus, it is suggested that yayurea A and B keep the *V. harveyi* in a phenotypic state of low cell density, although the cells have grown to high density.

When both AI-1 and yayurea A or B were applied, HAI-1 overruled the effect of yayurea A and B *in vitro*. Bioluminescence of the wild type strain *in vivo* significantly decreased after exposure to yayurea A and B, although the effect of the latter compound was smaller. Obviously, HAI-1 did not overrule the effect of yayurea A and B *in vivo*. This difference can be explained by the fact that bioluminescence was measured in stationary phase grown cells at a time when the impact of HAI-1 on QS induction is low [49]. Furthermore, the contribution of the other receptors (LuxQ and CqsS) and their cognate autoinducers (AI-2 and CAI-1) needs to be considered *in vivo* [49]. The ratio of the three receptors and the impact of their kinase and phosphatase activities on the output of the phosphorylation cascade is not yet fully understood. This could be another explanation for the strong effect of the quorum quenchers *in vivo*. Membrane-topology analysis predicts that LuxN is bound to the bacterial inner-membrane by nine transmembrane (TM) spanning domains [50], and periplasmic loop 3 might be the HAI-1 binding site [21]. We believe that yayurea A and B interfere with the AHL quorum sensing response of Gram-negative bacteria; since the tested bacteria on which the effects were observed have at least one AHL-based quorum sensing system (*Pseudomonas*, *Chromobacterium*, *Vibrio*, *and Serratia*).

Most natural environments harbor a stunningly diverse collection of microbial species. One example, is the marine bacterium *Halobacillus salinus*, which produces *N*-(2′-phenylethyl)-isobutyramide and 2,3-methyl-*N*-(2′-phenylethyl)-butyramide [51]. These compounds are unrelated to yayurea A (N-[2-(*1H*-indol-3-yl)ethyl]-urea) and B (N-(2-phenethyl)-urea). Within these communities, bacteria compete with their neighbors for space and resources [52]. Zoonotic commensals and pathogens, including yayurea producing staphylococcal species, use animals as a habitat. Whether these animals are also colonized by Gram-negative bacteria, other than in the gut, has barely been investigated. However, one can assume that animals bathe in puddles, lakes, and rivers, suggesting that their mucus, skin, fur, or feathers may easily encounter Gram-negative bacteria, being transient to permanent colonizers. One can also assume, that the ‘intermedius group’ share the habitat animal with Gram-negative bacteria. *S. delphini* was isolated from dolphin. Since both *V. harveyi* and *S. delphini* are marine bacteria and animal pathogens, it is conceivable that both bacteria share the same habitat and moreover, the same host surface. Our results demonstrated suppression of the quorum sensing regulated bioluminescence of *V. harveyi* during co-culture with *S. delphini* (Fig. 1), indicating that yayurea A and B are effective quorum quenchers.

The *in vitro* phosphorylation assay gave first mechanistic insights on how yayurea A and B affect quorum sensing of *V. harveyi*. We detected a significant and specific effect of yayurea A and B on the AHL-receptor LuxN. The concentrations used for these *in vitro* assays might not be physiological, especially when the influence of both molecules (HAI-1 and yayurea) was studied. Furthermore, it should be noted that HAI-1 is not constantly produced [49]. Therefore, in the natural habitat there might be times, when yayurea from *S. delphini* can fully interact with LuxN from *V. harveyi* in the absence of any competition with HAI-1. It therefore makes sense that the zoonotic staphylococci impair the QS-system of Gram-negative bacteria for competitive reasons. The growth of these staphylococci is not impaired by *P. aeruginosa* because the excreted yayureas suppress the production of the QS-controlled toxins and are thereby protected from being killed by the toxins (Fig. 3). While biofilm formation appears to be modulated by many regulators and environmental conditions in *P. aeruginosa*, pyocyanin and cyanide are controlled by the Las-QS system [53], implying that yayurea A and B might compete with 3-oxo-C12-HSL for LasR interaction.

When yayurea A (125 µg/ml) was added, the growth of *P. aeruginosa* was not affected (Fig. 6A), but quorum sensing and biofilm formation (Figs. 6A and 5C) were inhibited by 20%, which indicates that biofilm inhibition takes place prior to the onset of growth inhibition. In addition to their quorum quenching activity in Gram-negative bacteria, yayureas also inhibited their growth at higher dose (Fig. 2), which is a further advantage in the race for space and resources. For this advantage the zoonotic staphylococci are apparently prepared to pay a certain price, namely the production and excretion of comparatively high amounts of yayurea A and B. However, the benefit in competitiveness appears to prevail the cost disadvantages. As QS controls not only virulence factors but also many metabolic functions important for fitness, we don't know whether the inhibition of growth is a consequence of QS-inhibition or vice versa [54]–[56]. For some antibiotics (azithromycin, ceftazidime, and ciprofloxacin), it has been shown that they decrease the expression of QS-regulated virulence and many other genes [57].

The yayureas are potential candidates for use as anti-infectives. The only disadvantage might be the rather high concentration needed to completely quench QS; on the other hand, preliminary results suggest that they hardly have cytotoxic activities.

**Figure 8:**
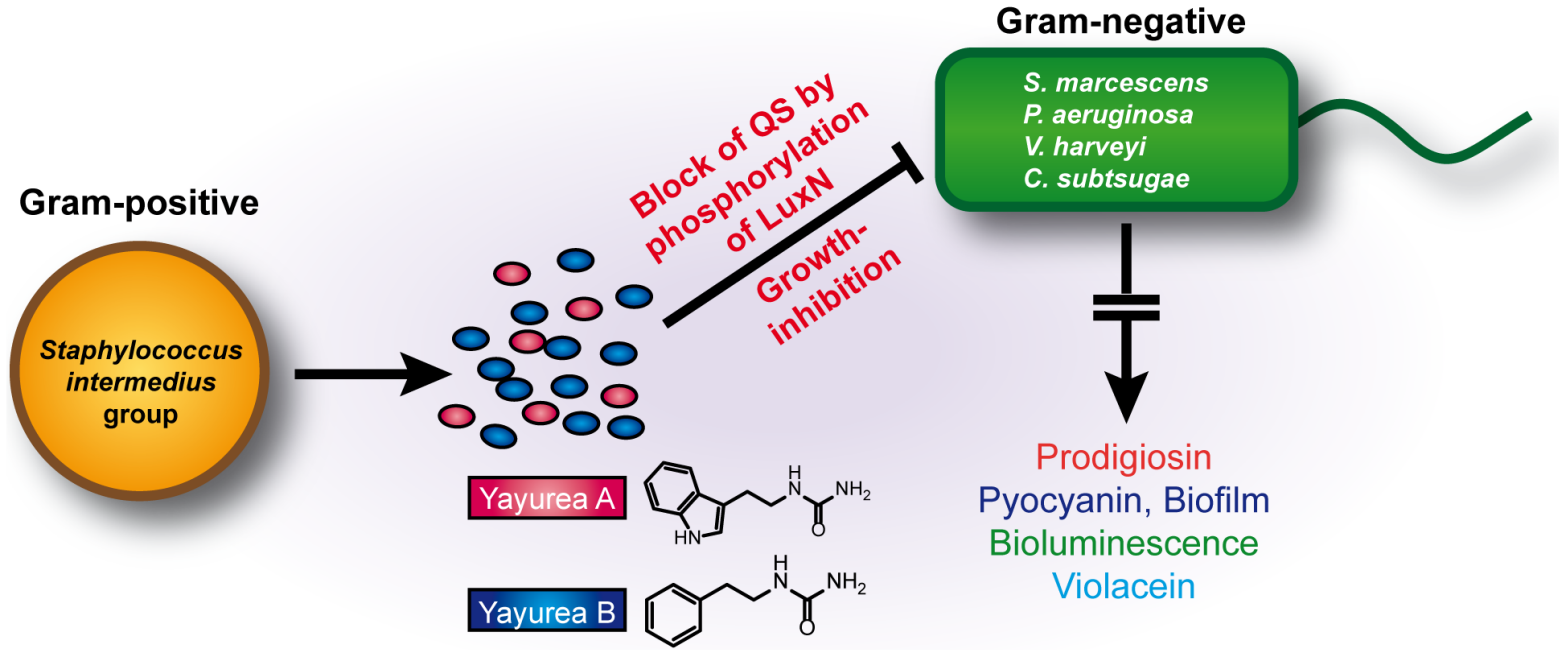
Schematic presentation of the interference between zoonotic staphylococcal species and Gram-negative bacteria. Animal associated (zoonotic) *Staphylococcus* species excrete novel quorum-quenching compounds, yayurea A and B, which block quorum sensing system in various Gram-negative bacteria by activating LuxN phosphorylation and have also growth inhibiting activity. The benefit for the staphylococcal species is better survival and increased competitiveness in a joint ecosystem.

An interesting question is why just the ‘intermedius group’ is producing yayureas. The most likely answer is that this group shares its habitat with Gram-negative bacteria. There must be a benefit to producing yayurea A and B because they are excreted in such high amounts certainly costing energy. In addition, the yayureas show growth inhibition only at quantities that exceed the physiological concentrations. It is a clever arrangement, that *S. delphini* produces just enough yayurea A and B to almost completely silence the expression of the studied QS-regulated compounds or biofilm formation in diverse Gram-negative bacteria. In mixed cultures, the yayurea-producing staphylococci arrest Gram-negative bacteria in a pheno- and genotypic state of low cell density, although the cells have grown to high density. The advantage for the staphylococci is twofold, on one hand, they are protected from QS-controlled toxins and on the other hand, yayurea A and B affect stationary growth of particularly those Gram-negative bacteria with prominent QS-control systems. This is one of the rare cases of inter-phylum interference between firmicutes (Gram-positive) and beta-/gammaproteobacteria (Gram-negative). A schematic presentation of the interference is shown in Fig. 8.

## Materials and Methods

### Bacterial strains and growth conditions

*Staphylococcus* strains were grown in Tryptic Soy Broth (TSB, Sigma) medium at 37°C, *Vibrio cholerae* SP27459 (O1 El Tor), *Serratia marcescens*, *Chromobacterium subtsugae* DSMZ 17043 and *Pseudomonas aeruginosa* were grown in lysogenic broth (LB) medium, and *Vibrio harveyi* DSMZ6904 in marine broth (MB; 5 g peptone, 3 g yeast extract and 75% sea water per liter of deionized water) at 28°C. The *Staphylococcus* strains tested in this work are listed in Table 2.

### Mixed cultivation of *Staphylococcus sp.* with *P. aeruginosa*

In co-culture experiments, we inoculated TSB with *S. aureus* (OD_578_ 0.005) and *P. aeruginosa* SH1 (OD_578_ 0.005). For the protection test, yayurea A (100 µg/ml), yayurea B (900 µg/ml), or water (control) were added to the mixed culture of *S. aureus* and *P. aeruginosa* after 3 h incubation. For co-cultivation of *S. delphini* with *P. aeruginosa*, each of the strains was inoculated with OD_578_ 0.001, because doubling time of *S. delphini* was shorter than that of *S. aureus*. Co-cultures were aerobically grown at 37°C. For CFU determination, samples were diluted and plated on Chapman agar (selective medium for staphylococci, while growth of *P. aeruginosa* is retarded) and BM agar (1% tryptone, 0.5% yeast extract, 0.5% NaCl, 0.1% K_2_HPO_4_, 0.1% glucose) on which *P. aeruginosa* grows better than the staphylococci. Colonies were counted after incubating for 24 h at 37°C; staphylococcal and pseudomonas colonies could be easily distinguished by colony shape and pigmentation.

### Purification and structural analysis of the QS-inhibiting compounds from *S. delphini*

The QS inhibitors were isolated from aerobically-grown overnight culture of *S. delphini* (vigorous shaking at 37°C, 20 h). Cells were centrifuged and the supernatant was applied to an Amberilite XAD 16 resin (Rhom& Haas, Germany). The column was first washed with water, then with 40% and 60% methanol and finally eluted with solvent A (80% methanol containing 5% acetic acid). Yayureas were significantly enriched in the last fraction. In the second purification step, the eluate was evaporated *in vacuo*, suspended in water, and further separated on an Amberilite IRC 50 cation exchange resin (Serva, Heidelberg, Germany) after the pH was adjusted to 7.0 with 1 M NaOH. The column was washed with water, then 70% ethanol and eluted with solvent A. The eluate was concentrated *in vacuo*, diluted, and adjusted to a final concentration of 50 mM sodium phosphate buffer with a pH of 4.2. In the third purification step, the eluate was separated on a SP Sepharose cation exchange column (GE Healthcare, Germany) with a linear 1 M NaCl gradient in 50 mM sodium phosphate buffer on a Äkta FPLC (GE Healthcare, Germany). The two active compounds were eluted separately at 120 mM and 280 mM NaCl concentration respectively. The final purification and desalting of each peak was carried out by reversed phase preparative HPLC (RP-HPLC) (Bischoff, Leonberg, Germany) on a nucleosil 100 C-18, 8×250 mm column (Machery Nagel, Düren, Germany) with a linear water acetonitrile (containing 0.1% TFA) gradient of 0% to 60% in 25 min. Purified compounds were lyophilized and stored at −20°C. Qualitative analysis was carried out on an Agilent 1200 HPLC system (Agilent technologies, Waldbronn, Germany) and a RP-HPLC Waters xBridge C18, 5 mm, 4.6×150 mm column. Compounds were eluted with a 15 min linear gradient of 0.1% phosphoric acid to acetonitrile at a flow rate of 1.5 ml/min. For structure elucidation, mass spectrometry was carried out on GC-MS and FT-ICR MS (Bruker, ApexII). NMR spectra were measured in d_4_-methanol and recorded on a Bruker AMX600 spectrometer (600 MHz for ^1^H, 150 MHz for ^13^C), solvent was used as internal standard (δ_H/C_ 3.31/49.15 for MeOH-*d_4_*).

### Assessing QS-regulated compounds in *S. marcescens*, *P. aeruginosa*, *C. subtsugae*, and bioluminescence in *V. harveyi* in co-cultivation with *Staphylococcus sp.*

Overnight cultures of *S. aureus* and *S. delphini* cells were diluted in LB medium containing 0.3% glucose to an OD_578_ value of 1.0 (we also tried 0.1, 0.5, but OD 1.0 results were most pronounced), incubated for 4 h at 37°C, then co-cultivated at 30°C with *S. marcescens* or *P. aeruginosa*; each strain was inoculated with a starting OD_600_ of 0.01. After 24 h of cultivation, prodigiosin of *S. marcescens* was extracted from the cell pellets by ethanol acidified with 4% of 1 M hydrochloric acid and then quantified by A_534 nm_ determination. Pyocyanin in the supernatant was determined by its absorption maximum at 520 nm [58]. For bioluminescence tests, overnight-cultured *V. harveyi* was diluted to an OD_578_ of 1.0 and co-cultivated with an equal amount of *S. aureus* or *S. delphini* cells in LB-MB (LB medium containing 0.1% glucose mixed with equal volumes of MB) for 18 h. Overnight cultures of *S. aureus* and *S. delphini* cells were diluted to an OD_578_ of 0.1, incubated for 4 h at 37°C, then co-cultivated at 30°C with *C. subtsugae*, which was diluted to a final OD_600_ value of 2; this high OD was necessary to pronounce violacein production, whose expression is dependent on AHL at higher cell density. After 24 h of incubation, cell pellets were collected and resuspended in water. Cells were lysed by 10% sodium dodecyl sulfate and incubated for 5 min at room temperature. Violacein was quantitatively extracted from the cell by adding water-saturated butanol. The butanol phase containing the violacein was collected and determined at its absorption maximum at 585 nm.

### Production of yayurea A and B in *S. delphini* and their effect on bacterial growth

*S. delphini* was inoculated to a starting OD_578_ of 0.1 and incubated at 37°C. During the 24 h incubation, cell density was followed by OD_578_ and the active compounds in the supernatants were determined by RP-HPLC and phosphoric acid – acetonitrile gradient as described above. The Gram-negative representatives, *E. coli*, *P. aeruginosa*, *S. marcescens*, *V. cholerae* and *V. harveyi* were inoculated to OD_600_ of 0.1 and grown in LB for 2 h. After that time, 1 mg/ml yayurea A or B (as solution in H_2_O) or an equal volume of water as negative control was added. Cells were either incubated at 37°C (*S. aureus* or *S. delphini*) or 30°C (*E. coli*, *S. marcescens*, *V. harveyi*, *V. cholerae* and *P. aeruginosa*). Cell density was followed for 24 h.

### Activity assays for QS-regulated compounds, bioluminescence, and biofilm formation in Gram-negative bacteria

#### Pyocyanin production

An overnight culture of *P. aeruginosa* PAO1 was diluted with LB medium to an OD_600_ of 0.1. The (Z-)-4-*bromo-*5-(bromomethylene)-2(5H)-furanone (Sigma), yayureas and tetracycline were dissolved in 10% DMSO. 190 µl diluted PAO1 was cultured in 96-well plates with 10 µl different concentrations of pure yayurea A, B, furanone, or 10% DMSO (negative control) at 30°C. After 24 h of incubation, cell density was measured at 600 nm and pyocyanin was isolated as described previously [58]. To measure pyocyanin production, the supernatants were subjected to HPLC separation on a Nucleosil 100, C-18 column and a 0–100% Water-ACN Gradient (water containing 0.1% phosphoric acid) in 15 min at a flow rate of 1.5 ml/min. The content of pyocyanin was determined at its absorption maximum at 520 nm [58]. The relative pyocyanin production was calculated as the ratio of the amount of pyocyanin content from the HPLC measurements to cell density.

#### Prodigiosin production

*S. marcescens* cells were inoculated from an overnight culture to a starting OD_600_ of 0.1. Then 100 µl of the diluted culture was mixed with 100 µl of different amounts of the purified compounds or water in wells of 96-well microtiter plates and incubated for 24 h at 28°C. Cell density was measured at 600 nm. Prodigiosin was extracted as described above and the relative prodigiosin production was calculated as the ratio between the amount of prodigiosin at its absorption maximum at 534 nm and cell density values at 600 nm. The prodigiosin suppressing effect of yayurea A and B was determined (H_2_O was used as control).

#### Biofilm formation assay

Biofilm formation assays were performed as described previously [34]. A full-grown culture of *P. aeruginosa* PAO1 or *S. aureus* was diluted with LB medium containing 0.3% (w/v) glucose to an OD_600_ of 0.1. 100 µl of the dilution was mixed with 100 µl of the yayurea solutions with the indicated concentrations, and incubated in 96-wellplates at 37°C for 24 h. To determine the biofilm formation, planktonic cells were discarded and the plates gently washed with PBS and air-dried for 30 min. The wells were stained with 200 µl of a 0.1% crystal violet solution at room temperature for 30 min. The stained biofilm was rinsed with distilled water followed by the addition of 200 µl DMSO. Absorbance of crystal violet as indicator for biofilm-forming bacteria was measured at 590 nm. The effects of the compounds were evaluated for which the sample with water was set to 100%.

#### Bioluminescence

An overnight culture of *V. harveyi* was diluted to an OD_600_ of 0.1 and grown with yayurea A, B, or water for 24 h at 28°C. Bioluminescence was determined in a Tecan infinite M200 plate reader (Tecan, Groedig, Austria). Relative luminescence was normalized by the OD_600_ values. The effects of the compounds were calculated by the relative luminescence, for which the sample with water was set to 100%.

### Investigation of the QS-target(s) of yayureas *in vitro*

#### Production of QS-receptors and QS-molecules

Inverted membrane vesicles were prepared using *E. coli* strainTKR2000 expressing plasmids pNKN, pNKQ, and pNKS encoding wild type LuxN, LuxQ, and CqsS (each with a C-terminal His-tag), respectively. Inverted membrane vesicles were prepared as described by [36]. LuxP was produced in, and purified from, *E. coli* MDAI-2 transformed with the plasmid pGEX_LuxP as described before [59]. LuxU was produced and purified as described before, using *E. coli* JM109 transformed with plasmid pQE30LuxU-6His [36]. Synthetic autoinducer, HAI-1, (from the University of Nottingham) was dissolved in a minimal volume of acetonitrile [10% (v/v)], diluted with water to a concentration of 100 mM and stored at −20°C.

#### Phosphorylation assays

Each QS-receptor kinase was tested as full-length membrane integrated proteins in inverted membrane vesicles. The buffer used for phosphorylation reactions was as follows: 50 mM Tris/HCl pH 8.0, 10% (v/v) glycerol, 500 mM KCl, 2 mM DTT. Each phosphorylation reaction contained equimoloar receptor concentrations [the total protein concentration were accordingly adapted: 3.2 mg/ml (LuxN), 5.5 mg/ml (LuxQ), and 5.2 mg/ml (CqsS) membrane proteins], and purified LuxU at 0.1 mg/ml. For phosphorylation of LuxQ, LuxP was added at a concentration of 0.4 mg/ml. LuxP integration into LuxQ-bearing membrane vesicles was triggered by several cycles of thawing and freezing. When indicated, yayurea A or B were added to the reaction mix at a final concentration of 220 µg/ml, and HAI-1 at 20 µM. The reaction was started by adding radio labeled Mg^2+^-ATP, typically 100 µM [γ-^32^P]ATP (specific radioactivity of 0.94 Ci/mmol; Perkin Elmer) and 110 µM MgCl_2_. The reaction was stopped after 1 and 10 minutes by adding SDS Laemmli-loading buffer followed by separation of the proteins on SDS-polyacrylamide gels. Gels were dried at 80°C on filter paper, exposed to a phosphoscreen for at least 24 h, and subsequently scanned using a PhosphorImager SI (GE Healthcare). The gels presented are representative of at least three independent sets of experiments.

## Supporting Information

Figure S1 **Mass spectra of Yayurea A and B. Mass spectrometry was carried out on GC-MS and FT-ICR MS (Bruker, ApexII).**
(TIF)

Figure S2 **Influence of yayurea A and B on growth.** Growth curve of *S. marcescens*, *P. aeruginosa*, *V. harveyi* and *V. cholerae* in BM with 1000 µg/ml yayurea A or B or equal volume of water. Arrow indicates time point (after 2 h) of the addition of compounds to the growing culture. All the measurements were made in triplicate. Bars indicate standard deviation, SD.
(TIF)

Figure S3 **Phylogenetic tree among** ***Staphylococcus*** **species.** The tree is based on 16S rRNA relationships according to [45]. The phylogenetic position of the ‘intermedius group’ composed of *S. intermedius*, *S. pseudintermedius*, and *S. delphini* is marked in bold. Based on a combinatin of 16S rRNA and multilocus data, the group was recently complemented by the next related species *S. lutrae*, *S. schleiferi* subsp. *schleiferi* and *S. schleiferi* subsp. *coagulans* [37]. All members of this group produce yayurea A and B.
(TIF)
