# Supplementary figures and images for: A New Class of Quorum Quenching Molecules from Staphylococcus Species Affects Communication and Growth of Gram-Negative Bacteria
Source: PLoS Pathog. 2013 Sep 26;9(9):e1003654. doi: 10.1371/journal.ppat.1003654 (PMC3784491; doi:10.1371/journal.ppat.1003654)

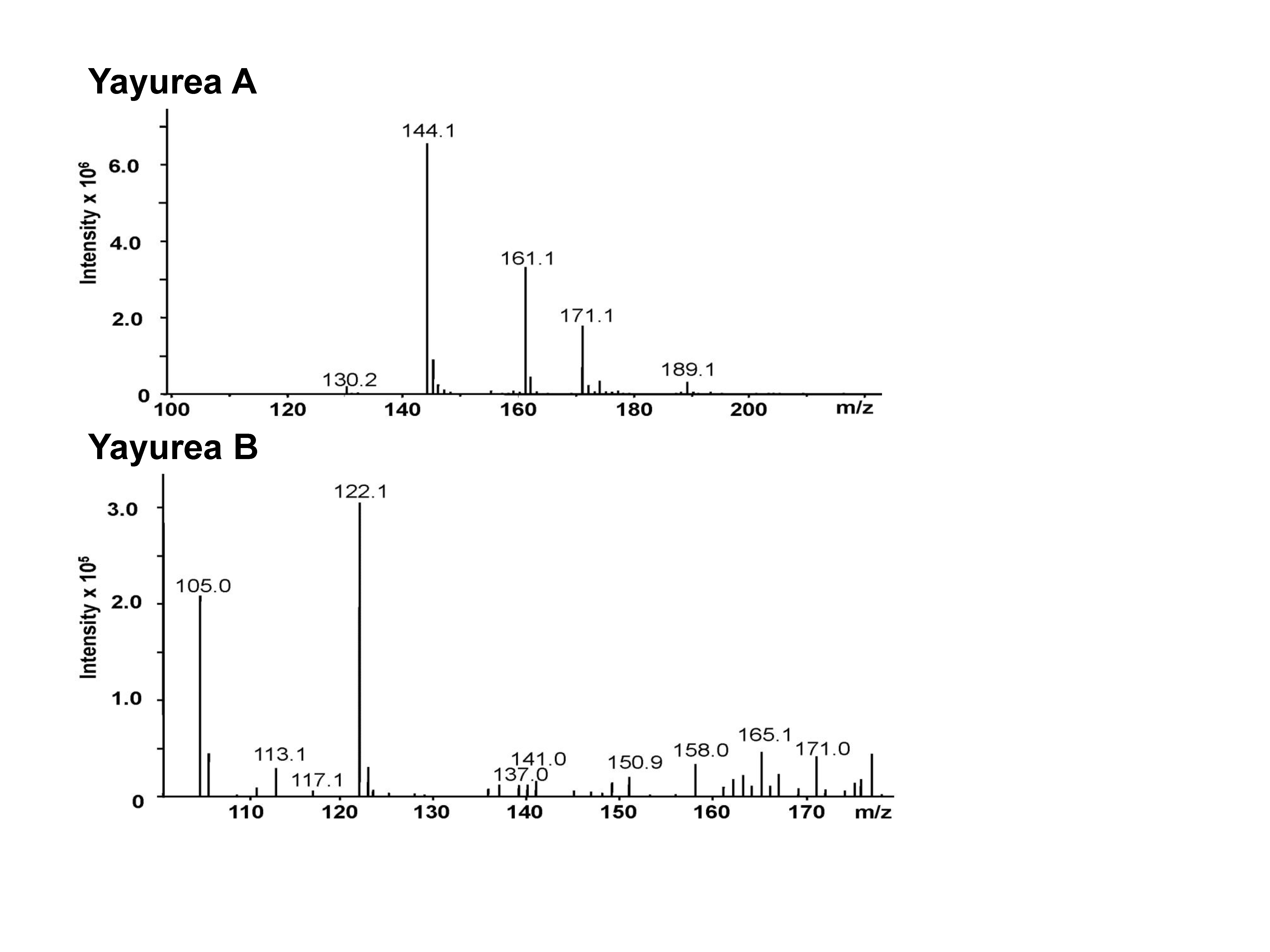

Supplement: Figure S1 — Mass spectra of Yayurea A and B. Mass spectrometry was carried out on GC-MS and FT-ICR MS (Bruker, ApexII). (TIF) [file ppat.1003654.s001.tif]
